# Epigallocatechin-3-Gallate Ameliorates Glucocorticoid-Induced Osteoporosis of Rats *in Vivo* and *in Vitro*

**DOI:** 10.3389/fphar.2018.00447

**Published:** 2018-05-09

**Authors:** Shengye Liu, Liyu Yang, Shuai Mu, Qin Fu

**Affiliations:** Department of Spine and Joint Surgery, ShengJing Hospital of China Medical University, Shenyang, China

**Keywords:** dexamethasone, epigallocatechin-3-gallate, glucocorticoid-induced osteoporosis, reactive oxygen species, Nrf2

## Abstract

**Background:** Prolonged administration of overdoses of glucocorticoids results in increased bone remodeling, leading to glucocorticoid-induced osteoporosis (GIO), which is primarily due to the dysfunction and apoptosis of osteoblasts. The present study investigated the therapeutic effect and molecular mechanism of action of epigallocatechin-3-gallate (EGCG), a bioactive catechin in green tea, in high-dose dexamethasone-induced osteoblast differentiation *in vivo* and *in vitro*.

**Methods:** The anti-dexamethasone (DEX) effects of EGCG on primary osteoblasts were determined on the basis of cell viability and alkaline phosphatase (ALP) and total cellular superoxide dismutase (SOD) activities. Flow cytometry and Western blot analysis were also used to evaluate the expression of related biomarkers *in vitro*, and bone microarchitecture was also extensively examined in a rat model *in vivo*.

**Results:** The results showed that EGCG pretreatment significantly increased osteoblast viability and ALP and SOD activities when cells were exposed to DEX. Alizarin red staining indicated that there was more mineralization with EGCG pretreatment, countering DEX effects. EGCG reduced DEX-induced reactive oxygen species at both the mitochondrial and cellular levels in osteoblasts by activating the nuclear factor erythroid-derived 2-like-2 (Nrf2) pathway. In addition, EGCG protected osteoblasts from apoptosis. EGCG also regulated the formation of active glucocorticoid by 11β-hydroxysteroid dehydrogenase activity. Furthermore, femoral micro-computed tomography scans revealed that EGCG improved bone microstructure and mitigated DEX-induced deterioration of bone quality.

**Conclusion:** These findings suggested that EGCG reversed GIO in rats by protecting osteoblasts by activating the Nrf2 signaling pathway.

## Introduction

Glucocorticoid-induced osteoporosis (GIO) is one of the most common forms of secondary and iatrogenic osteoporosis. Glucocorticoids in excess exert their effects mainly on osteoblasts, which are essential for bone formation. Dysfunction and apoptosis of osteoblasts caused by dexamethasone (DEX) have been identified as a considerable contributor to the development of GIO (Li et al., 2015).

Glucocorticoid action on target tissues is determined by the density of “nuclear” receptors and intracellular metabolism by the two isozymes of 11β-hydroxysteroid dehydrogenase (11β-HSD). They metabolize glucocorticoids at the pre-receptor level and can thus control intracellular concentrations of active glucocorticoids (Stewart and Krozowski, 1999). This intracellular glucocorticoid availability is established by the interconversion of hormonally active and inactive ligands controlled by two types of 11β-HSD. 11β-HSD type 1 (11β-HSD1) is widely expressed in liver, adipose tissue, muscle, pancreatic islets, adult brain, inflammatory cells, and gonads. Previous studies have shown that the effects of glucocorticoids on bone are dependent on the autocrine actions of 11β-HSD1 (Cooper et al., 2002), whose expression in osteoblasts fosters the local synthesis of active glucocorticoids and leads to increased intracellular concentrations of active glucocorticoids (Seibel et al., 2013). On the other hand, 11β-HSD type 2 (11β-HSD2) is a high-affinity dehydrogenase and predominantly catalyzes the formation of inactive corticosterone from active cortisone, and 11β-dehydrocorticosterone reversely by 11β-HSD1. Bone formation is decreased by excess glucocorticoids while bone resorption is enhanced, leading to osteopenia and ultimately osteoporosis. To amplify the abundance of 11β-HSD2 and control 11β-HSD1 is an effective way to suppress excess active glucocorticoids. The regulation of 11β-HSD activity conversion is a target aimed at protecting against bone loss due to excess intracellular glucocorticoids.

Epigallocatechin-3-gallate (EGCG) is a bioactive constituent accounting for more than 50% of the total catechins in green tea, whose benefits have been revealed by many epidemiological investigations (Harborne and Williams, 2000; Song et al., 2014). It has been associated with antiaging properties, improved redox status, antitumor and anti-Alzheimer activity, etc. (Afzal et al., 2015). Besides, Hintzpeter et al. (2014) have provided evidence that the activity of the polyphenolic EGCG may be attributed to strong inhibition of the cortisol-producing enzyme 11β-HSD1, which made us wonder whether it could be regarded as a target of GIO. Reactive oxygen species (ROS) induced by excess glucocorticoids in cells would also result in the dysfunction or even apoptosis of osteoblasts and severely interfere with osteogenic differentiation. All these factors contribute to the disorder of bone formation and incidence of osteoporosis.

Epigallocatechin-3-gallate is a polyphenol well known for its antioxidant properties. Previous studies have shown that EGCG can act as a pro-osteogenic agent to enhance osteogenic differentiation of mesenchymal stem cells, suppress osteoclast differentiation, and reduce bone resorption (Jin et al., 2014; Tominari et al., 2015). However, it is unknown whether EGCG’s protective potential is applicable in protecting against GIO by reducing ROS and improving cellular function. On the basis of the evidence above, our study aimed to explore the protective mechanism of EGCG in primary osteoblasts and its therapeutic effect in a GIO model.

## Materials and Methods

### Reagents

Purified EGCG and DEX (>98%; Sigma-Aldrich; St. Louis, MO, United States) were stored at -20°C. Dulbecco Minimum Essential Medium (DMEM, high glucose) and trypsin-EDTA were obtained from GE Healthcare Life Sciences (Hyclone; Logan, UT, United States). Cell Counting Kit-8 (CCK-8) was purchased from Sigma-Aldrich (St. Louis, MO, United States). Reactive Oxygen Species Assay Kit and Annexin V-FITC Apoptosis Detection Kit were purchased from Beyotime Institute of Biotechnology (Jiangsu, China). MitoSOX^TM^ Red mitochondrial superoxide indicator was from Sigma-Aldrich (St. Louis, MO, United States), rabbit anti-PARP (No. PB0343) was purchased from Boster Biotechnology, Inc., and rabbit anti-cytochrome C (Cat. No. ab13575), rabbit anti-HO-1 (Cat. No. ab68477), rabbit anti-nuclear factor erythroid 2-related factor 2 (Nrf2; Cst. No. 12721), and mouse anti-β-actin (Cat. No. ab8226) monoclonal antibodies were purchased from Abcam (Cambridge, MA, United States) and CST (Cell Signaling Technology, Inc.). Primers were designed and synthesized by Sangon Biotech Co., Ltd. (Shanghai, China). Invitrogen TRIzol reagent was obtained from Thermo Fisher Scientific, Inc. (Waltham, MA, United States).

### Isolation and Culture of Primary Osteoblasts

Primary osteoblasts were isolated from neonatal rats as described previously (Shim et al., 2016). Cells were resuspended and maintained in DMEM high glucose supplemented with 20% fetal bovine serum (FBS; PAN-Biotech, Adenbach, Germany), 100 U/mL penicillin, and 100 μg/mL streptomycin, in a 5% CO_2_ humidified atmosphere at 37°C.

### Cell Viability After EGCG and DEX Treatments

The CCK-8 assay was carried out for measuring cell proliferation. Briefly, osteoblasts were seeded at a density of 5 × 10^3^ cells/well in 96-well plates and incubated overnight. The DEX group was treated with 100 μM DEX, while the EGCG-treated group was pretreated with 5 μM EGCG for 2 h and then exposed to 100 μM DEX for 24 h. Subsequently, 10 μL of CCK-8 reagent was added to each well and incubation was continued for 2 h. Absorbance was read at 450 nm on a microplate reader to determine cell viability.

### ALP Activity Assay

To determine alkaline phosphatase (ALP) activity, osteoblasts were incubated in different conditioned media in 6-well plates at a density of 1 × 10^5^ cells/well for 7 days. After cells were lyzed with 100 μL of assay lysis buffer, the ALP activity levels were determined with an ALP reagent kit (Nanjing Jiancheng Bioengineering Research Institute, Nanjing, China) following the manufacturer’s instructions. Lysis concentration was adjusted for assays in a 96-well plate. ddH_2_O, 5 μL, was added to the control well and 5 μL of phenol application liquid was used in the standard well. Together with control and standard well, lysis samples were supplemented with 50 μL of buffer and 50 μL of matrix liquid. The plate was then incubated at 37°C for 30 min, and 150 μL chromogenic agent was added to all wells and the plate gently mixed. Absorbance was read at 520 nm on a microplate reader and ALP activity was calculated.

### Analysis of Total Cellular Superoxide Dismutase (SOD) Activity

Cell pretreatment was the same as previously indicated in the CCK-8 assay. Total superoxide dismutase (SOD) activity was measured using a Total Superoxide Dismutase Assay Kit with NBT (S0109, Beyotime) following the manufacturer’s protocol. After 24-h treatment with DEX and EGCG, osteoblasts were washed twice with cold PBS, then lyzed in PBS by pulse sonication on ice, and subsequently centrifuged at 13,000 *g* at 4°C for 10 min. The supernatant was then transferred to a fresh tube. The sample protein concentration was measured and adjusted to 1 μg/μL. A 20 μL volume of the sample or SOD assay buffer (blanks) was added to a 96-well plate, along with 160 μL of NBT/enzyme working solution and 20 μL of reaction initiation working solution, to all wells except for Blank2. The plate was incubated at 37°C for 30 min. SOD activity was calculated after reading absorbance at 560 nm on a microplate reader.

### Flow Cytometric Analysis of Cellular Reactive Oxygen Species and Mitochondrial Superoxide Production

Total amount of intracellular ROS was measured with the non-fluorescent 2,7-dichlorofluorescin diacetate (DCFH-DA) probe, and mitochondrial superoxide was assayed by a MitoSOX^TM^ Red mitochondrial superoxide indicator. The treated cells were harvested and rinsed with PBS, and then incubated in 10 μM DCFH-DA for 20 min or 5 μM MitoSOX^TM^ Red florescent probe for 10 min at 37°C, followed by washing with warm buffer. Fluorescence was detected and analyzed by FACScan flow cytometry (Becton Dickinson, Franklin Lakes, NJ, United States).

### Mineralization and Alizarin Red Staining

Primary osteoblasts were cultured on 35-mm dishes in DMEM high glucose medium while replenished every other 2 days. After 14 days of culture, cells were fixed in 90% ethanol at room temperature for 30 min. Cells were stained with 1 mL of 40 mM Alizarin Red-S (pH 4.2) at room temperature for 20 min while being gently mixed. The cells were washed sufficiently with 2 mL of distilled water to avoid non-specific staining so that mineralized nodules and stained cells could be visualized and photographed under a microscope (Zeiss, Oberkochen, Germany).

### Osteoblast Apoptosis Detection

Osteoblasts were incubated in a 6-well plate at a density of 2 × 10^5^ cells/well. The cells were exposed to 100 μM DEX with or without pretreatment with EGCG for the indicated time periods until they were harvested, and then resuspended in 500 μL of binding buffer containing 5 μL of Annexin V-APC and 5 μL of PI for 20 min. After being rinsed twice with PBS, cells were placed in an ice bath. Afterward, all samples were subjected to FACScan flow cytometry (Becton Dickinson, Franklin Lakes, NJ, United States).

### Gene Expression by Real-Time PCR

Cells were cultured in osteogenic induction medium with drug treatment for 2 days, and total RNA was then extracted with TRIzol reagent to synthesize cDNA using SuperScript II reverse transcriptase (Invitrogen; Thermo Fisher Scientific, Inc.) with 5 μg oligo (dT) primers per sample. By using SYBR Green PCR master mix (Applied Biosystems; Thermo Fisher Scientific, Inc.), qPCR was performed in a total volume of 20 μL in a 7500 Real-Time PCR System (Applied Biosystems; Thermo Fisher Scientific, Inc.) as follows: 95°C for 5 min, and 40 cycles of 95°C for 30 s and 60°C for 45 s. Melt-curve analysis was used to confirm the specificity of the amplification, and GAPDH served as the endogenous control for normalization of amount of total RNA in each group. The relative levels of gene expression were determined as ΔCq = Cq_gene_ – Cq_reference_, and gene expression was calculated as fold change according to the 2^-ΔΔCq^ method, while being repeated independently in triplicate. The primer sequences were designed as follows: forward, 5′-GAATGCACTACCCAGCCAC-3′ and reverse, 5′-TGGCAGGTACGTGTGGTAG-3′ for Runx2; forward, 5′-CTGACCACCTGAACTCCAC-3′ and reverse, 5′-CATCTAGGTACAACATGGAG-3′ for bone morphogenetic protein (BMP-2); forward, 5′-GTCAAGAGTCTTAGCCAAACTC-3′ and reverse, 5′-AAATGATGTGAGGCCAGATGG-3′ for Osterix; and forward, 5′-GTGAAGCAGGCATCTGAGGG-3′ and reverse, 5′-GCCGTATTCATTGTCATACCAGG-3′ for GAPDH.

### Western Blot Analysis

Total proteins were harvested in ice-cold radioimmunoprecipitation lysis buffer (Thermo Fisher Scientific, Inc.) supplemented with phenylmethanesulfonyl fluoride for 1 h. After protein concentration was assessed, equal proteins of each group were separated on 12% sodium dodecyl sulfate polyacrylamide (SDS-PAGE) gels and electrophoretically transferred onto polyvinylidene difluoride (PVDF) membranes (Millipore, Bedford, MA, United States). The membranes were blocked in 5% skim milk for 1 h. After being washed three times with Tris-buffered saline containing Tween-20, the membranes were incubated with primary monoclonal antibodies against Bcl-2, PARP, 11β-HSD1, 11β-HSD2, Runx2, Nrf2, or HO-1 overnight at 4°C followed by incubation with horseradish peroxidase (HRP)-conjugated secondary antibody. The relative protein levels were calculated on the basis of β-actin as the loading control. Signal detection was visualized by using enhanced chemiluminescence.

### Animals and Groups

Eighteen 8-week-old female experimental SD rats (weighing 245 ± 17 g) were obtained from the Animal Center of China Medical University. Rats were acclimated to specific pathogen-free laboratory conditions (a well-ventilated controlled room at 20°C on a 12-h light/dark cycle with free access to water and food) for 1 week prior to the drug treatments. Rats were evenly randomly distributed into three groups: control group, DEX groups, and DEX with EGCG (5 mg/kg/day). The GIO model was established by the intramuscular injection of 1 mg/kg/day DEX for 60 days. Control group was with equivalent normal saline administration. The EGCG-treated groups underwent 5 mg/kg/day EGCG by gavage with the same induction in the GIO model group. All animal care and experimental procedures were approved by the Institutional Animal Care Ethics and Use Committee of China Medical University and the number of protocol approval was 2016PS262K. We made every effort to minimize the animals’ suffering in accordance with the guidelines. Rats were euthanized and bilateral femurs were removed for further analysis.

### Immunohistochemistry

For immunohistochemistry, left femoral sections were prepared as previously described (Li et al., 2016), and they were then incubated overnight at 4°C with rabbit anti-cytochrome C. Rabbit serum (Solarbio, Beijing, China) was used as the blocking agent. The primary antibodies were detected after incubation with an anti-rabbit IgG secondary antibody conjugated with HRP for 30 min at 37°C. The results were visualized using a digital microscope (DP73; Olympus, Tokyo, Japan).

### Micro-Computed Tomography (Micro-CT)

Micro-computed tomography (micro-CT; QuantumGX, PerkinElmer, United States) was conducted on the proximal right femur to scan the microstructure of the femur, and parameters were analyzed precisely at the same region of interest (ROI) in cross section. Specimen scanner settings were designed as follows: exposure time 14 s at 90 kV and 88 μA with a resolution of 2 μm and field-of-view 12.8 mm × 12.8 mm. The structural parameters for trabecular bone were derived from micro-CT data, including trabecular separation (Tb.Sp; mm), trabecular number (Tb.N; mm^-1^), bone volume/tissue volume (BV/TV; %) trabecular thickness (Tb.Th; mm), connectivity density (Conn.D), and structure model index (SMI), which were evaluated on the basis of traditional static bone histomorphometry.

### Bone Mass Densitometry

Densitometry was performed by dual-energy X-ray absorptiometry (DXA) using a PIXImus II densitometer (GE Medical Systems, Lunar Division, Madison, United States) on right femurs and data were recorded. The measurement was limited to the proximal femur area of rats.

### Statistical Analysis

All the presented data and results were evaluated using GraphPad Prism 6.01 and were expressed as mean ±*SD* in at least three independent experiments. One-way analysis of variance was used to calculate the statistical variance. *P* < 0.05 (^∗^), *P* < 0.01 (^∗∗^) or *P* < 0.05 (#), *P* < 0.01 (##), and *P* < 0.001 (###) were considered statistically significant. Image Pro Plus software was utilized for analysis of Alizarin red staining and immunohistochemistry.

## Results

### EGCG Improves Osteoblast Cell Viability, ALP, and SOD Activities

**FIGURE 1:**
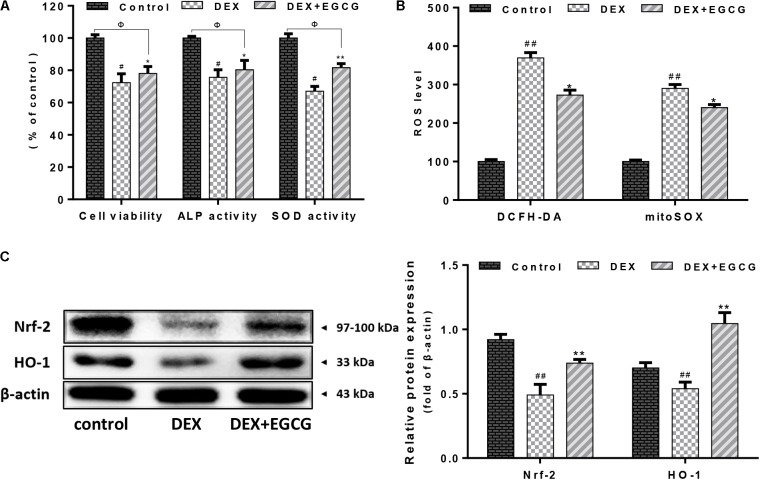
Effect of EGCG on DEX-induced osteoblast cellular dysfunction. **(A)** EGCG improved cell viability, increased ALP activity, and recovered SOD expression in primary osteoblasts treated with DEX. **(B)** EGCG reversed cellular and mitochondrial oxidative stress according to cytometry of DCFH-DA and mitoSOX staining. **(C)** Expression of Nrf2 and downstream transcriptional factor HO-1 during EGCG protection under high-dose DEX treatment. Protein levels were statistically evaluated in columns. Measurements were in triplicate and data are presented as the mean ±*SD*. ^##^*P* < 0.01, ^#^*P* < 0.05 vs. control; ^∗∗^*P* < 0.01, ^∗^*P* < 0.05 vs. DEX; and ^Φ^P < 0.05 DEX+EGCG vs. control.

As shown in **Figure 1A**, DEX decreased osteoblast cell viability and ALP and SOD activities, whereas pretreatment with EGCG prevented the DEX-induced changes in cell viability and cellular ALP and SOD activities, improving viability and cellular function.

### EGCG Activates Nrf2/HO-1 Signaling to Inhibit DEX-Induced Oxidative Stress

In **Figure 1B**, it was indicated that DEX increased ROS accumulation and suppressed antioxidant defense systems in osteoblasts. There was an evident increase in both mitochondrial ROS and intracellular ROS production in the DEX group. DEX increased DCFH-DA- and mitoSOX-positive signals by approximately threefold, compared to the control group. In contrast, pretreatment with EGCG reduced the increase in ROS levels and alleviated ROS damage. During this process, the Nrf2/HO-1 signaling pathway was activated and its protein expression was upregulated (**Figure 1C**).

### EGCG Inhibits DEX-Induced Apoptosis of Osteoblasts *in Vivo* and *in Vitro*

**FIGURE 2:**
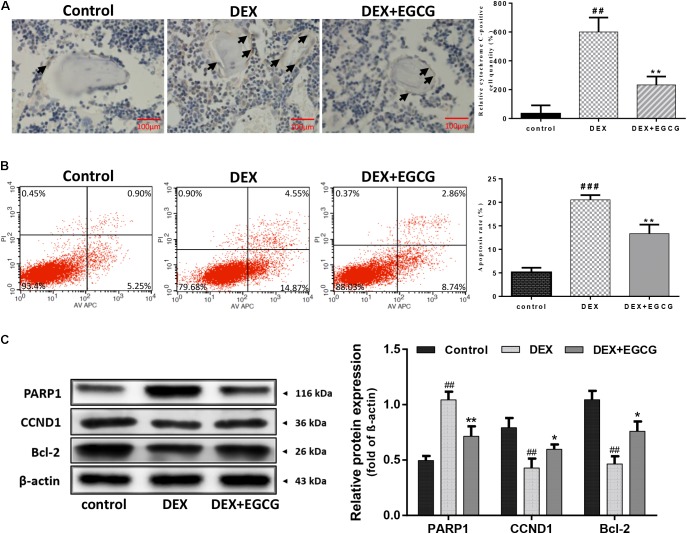
Protective effect of EGCG against DEX-induced apoptosis in GIO. **(A)** Cell apoptosis was measured by immunohistochemistry (arrow indicates cytochrome C positive cells) and was statistically evaluated. **(B)** Flow cytometric analysis was used to measure apoptotic rate, and the level of apoptotic rate was statistically evaluated, as shown in the column. **(C)** Expression of apoptosis-related protein was measured by Western blot, and quantitated levels of protein were statistically evaluated, as shown in the column. The data were expressed as the mean ± *SD*. ^###^*P* < 0.001, ^##^*P* < 0.01, ^#^*P* < 0.05 vs. control and ^∗∗^*P* < 0.01, ^∗^*P* < 0.05 vs. DEX.

The effect of EGCG on DEX-induced apoptosis and dysfunction of osteoblasts and femur tissues was determined by immunohistochemical assay. **Figure 2A** indicates that large numbers of cytochrome C-positive osteoblasts formed clusters around the trabecular bone in the DEX-induced osteoporotic group. Nevertheless, few cytochrome C-positive osteoblasts were observed in the control group and EGCG-treated group, suggesting that EGCG could rescue osteoblasts from DEX-induced apoptosis from a general perspective. To gain further insight into the effect of EGCG at the cellular level, Annexin V-APC/PI staining-based flow cytometry analysis was performed in primary osteoblasts. Approximately 20% of osteoblasts underwent apoptosis following exposure to DEX for 24 h compared to the control (**Figure 2B**). Particularly, pretreatment with 5 μM EGCG ameliorated DEX-induced apoptosis significantly, decreasing the high apoptotic rate induced by DEX. Meanwhile, as to protein level, exposure to DEX resulted in high PARP expression, which was also attenuated by EGCG pretreatment. Reduced bcl-2 and CCND1 could also be reversed by EGCG (**Figure 2C**). These findings demonstrated that EGCG could suppress DEX-induced apoptosis of osteoblasts and promote cell proliferation *in vitro* and *in vivo*.

### EGCG Reverses DEX-Induced Inhibition of Osteogenic Differentiation in Primary Osteoblasts

**FIGURE 3:**
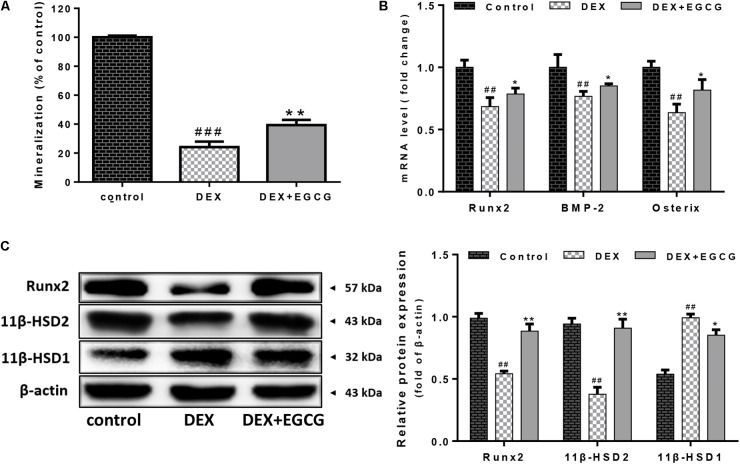
Effects of EGCG on DEX-induced differentiation and 11β-HSD activity in primary osteoblasts. **(A)** Mineralization of cells was inhibited by DEX, but pretreatment with EGCG reduced this effect. **(B)** Expression levels of representative osteogenic differentiation transcriptional factors (Runx2, BMP-2, and Osterix) were analyzed by real-time PCR. **(C)** Expression of 11β-HSD and Runx2 was measured by Western blot, and quantitated levels of protein were statistically evaluated, as shown in the column. The data were expressed as the mean ± *SD*. ^##^*P* < 0.01, ^###^*P* < 0.001 vs. control and ^∗∗^*P* < 0.01, ^∗^*P* < 0.05 vs. DEX.

We examined whether EGCG improved osteogenic differentiation in osteoblasts. Consistently, DEX reduced the formation of calcium deposits at a high concentration of 100 μM DEX. EGCG significantly reversed this effect and increased osteoblastic mineralization in cells treated for 14 days; mineralization formation ability was quatificated in **Figure 3A**. Moreover, as shown in **Figure 3B**, mRNA expression of some osteogenic marker genes, including Runx2, BMP-2, and Osterix, was also upregulated by pretreatment with EGCG, using real-time PCR. Runx2 protein expression by Western blotting was consistent with that of mRNA results (**Figure 3C**). These findings suggested that EGCG stimulated the maturation and differentiation of primary osteoblasts.

### EGCG Downregulates Synthesis of Active Glucocorticoids by Regulating 11β-HSD Activity

EGCG regulated the inter-conversion of 11β-HSD (**Figure 3C**). DEX increased the expression of 11β-HSD1 and reduced 11β-HSD2 expression. EGCG appeared to reverse the DEX-induced effects by enhancing 11β-HSD2 expression and also decreasing 11β-HSD1 expression.

### EGCG Reverses DEX-Induced Microstructure Destruction of Femoral Bone and Decrease in Bone Mineral Density of GIO Model

**FIGURE 4:**
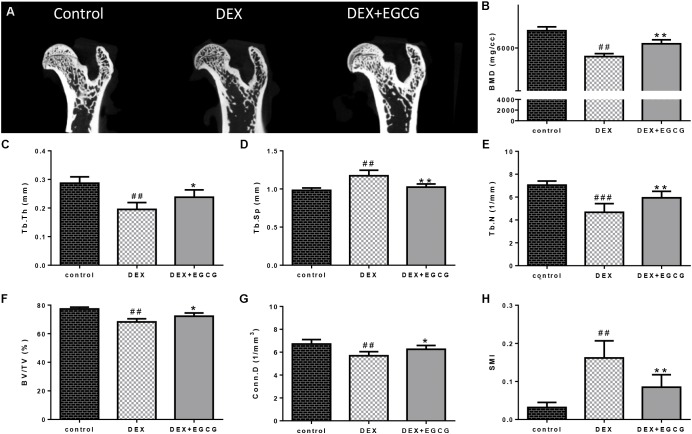
Effects of EGCG on trabecular bone micro-architecture in GIO rats. **(A)** micro-CT of proximal femurs. **(B)** Data from BMD measurements of femurs by DXA. The following computed tomographic indices were analyzed in the defined region of interest (ROI): **(C)** Tb.Th, **(D)** Tb.Sp, **(E)** Tb.N, **(F)** BV/TV, **(G)** Conn.D, and **(H)** SMI. The data were expressed as the mean ±*SD*. ^###^*P* < 0.001, ^##^*P* < 0.01, ^#^*P* < 0.05 vs. control and ^∗∗^*P* < 0.01, ^∗^*P* < 0.05 vs. DEX.

As illustrated in **Figure 4**, DEX caused damage to the microstructure of the proximal femoral bone. Some related parameters changed. Tb.Th, Tb.N, BV/TV, and Conn.D decreased, and Tb.Sp and SMI increased. Besides, bone mass density (BMD) was downregulated by DEX. Gavage with EGCG effectively improved bone quality by reversing the changes in these affected parameters.

## Discussion

Clinical glucocorticoid use is the leading iatrogenic cause of secondary osteoporosis (Caplan et al., 2017). Meanwhile, GIO may occur in 30–50% of patients undergoing glucocorticoid therapy (Weinstein, 2011). EGCG is the major component among the tea catechins and is believed to have a considerable therapeutic potential. In our study, we aimed to get full use of the natural bioactive component EGCG and evaluated it as a novel pharmacological agent for GIO. 11β-HSD1 and 11β-HSD2 are isoenzymes that catalyze the interconversion of hormonally inactive and active glucocorticoids. Increased fractures caused by glucocorticoid administration might be attributed to the increase in 11β-HSD1 (Cooper et al., 2002). 11β-HSD1 is selectively elevated in adipose tissue in obesity where it contributes to metabolic complications. Modulation of 11β-HSD1 activity in osteoblasts is being pursued as a new therapeutic approach for the treatment of GIO. Since EGCG was revealed to be a strong inhibitor of 11β-HSD1 activity (Hintzpeter et al., 2014). We wanted to prove that EGCG was also able to exert a similar effect in osteoblasts. Our results indicated that EGCG decreased 11β-HSD1 protein expression and increased 11β-HSD2 expression at the same time, which is beneficial for ameliorating GIO to some extent. EGCG was able to regulate glucocorticoid activity. The amplification of 11β-HSD2 and decline of 11β-HSD1 induced by EGCG facilitated the conversion of active glucocorticoid into the inactive form, which suggested that EGCG could be an antagonist against GIO by reducing active glucocorticoids.

It is generally accepted that ROS contribute to various pathological conditions that drive the irreversible destruction of cellular components, including DNA, organelles, and cytokines as well, resulting in cell apoptosis or necrosis (Sies, 2017). Studies have revealed that glucocorticoids initiate the generation of ROS (Lin et al., 2015). Earlier studies demonstrated that glucocorticoids could lead to ROS-induced apoptosis of osteoblasts in bone as well as decreased mineral deposition *in vitro* (Feng and Tang, 2014). Moreover, ROS were most derived from mitochondria. Incidentally, we detected both cellular and mitochondrial ROS production by DCFH-DA and mitoSOX fluorescence probes, which suggested that EGCG pretreatment reduced ROS levels and maintained cells in a stable state. Furthermore, SOD, an important antioxidant enzyme in cells, was upregulated by EGCG, which inhibited ROS production. Of the various cytoprotective systems, heme oxygenase-1 (HO-1) has been regarded as a stress enzyme involved in defense against agents that can induce oxidative damage (Jian et al., 2011). HO-1 protein expression is mediated by the transcription factor Nrf2 (Minelli et al., 2009). As indicated in **Figure 1C**, EGCG promoted the activation of Nrf2 and the subsequent induction of HO-1, tending to restore the intracellular balance between oxidants and antioxidants after DEX-induced oxidative insult.

Osteoblast apoptosis induced by glucocorticoids has been considered the critical factor in the pathogenesis of GIO (Sato et al., 2015; Corrado et al., 2017). The effect of EGCG on DEX-induced osteoblast apoptosis was further investigated by flow cytometry. On the one hand, Annexin V-APC/PI staining-based flow cytometry analysis showed that a large portion of DEX-treated osteoblasts underwent apoptosis, which was blocked by EGCG. On the other hand, the administration of EGCG in the GIO rat model produced a strong protective function in bone section. Cytochrome C is a pro-apoptotic molecule. Release of cytochrome C by activated mitochondria into the cytosol triggers caspase proteases, which mediates the mitochondrial apoptotic pathway. Immunohistochemical analysis demonstrated that EGCG decreased the number of cytochrome C-positive cells around bone matrix in the proximal femoral section shown in **Figure 2A**. Also, the expression of the pro-apoptosis protein PARP was downregulated, with the increased abundance of anti-apoptotic protein bcl-2. At the same time, CCND1 was upregulated compared to the DEX group, which indicated that EGCG could also enhance the proliferative activity of osteoblasts. These findings demonstrated that EGCG could protect osteoblasts from DEX-induced apoptosis.

Osteoblasts express extracellular matrix proteins such as ALP during the cell proliferation and mineralization phases. Its activity can directly reflect the status of osteoblast activity or function. Results in **Figure 1A** show that DEX decreased ALP activity by approximately 25% compared to the control, while pretreatment with EGCG retrieved ALP activity, indicating that EGCG promoted osteogenesis by improving phenotypic markers of ALP expression. Bone development is always accompanied by maturation of the extracellular matrix and mineralization (Yang et al., 2016). Ca^2+^ deposits for mineralization. Furthermore, we assessed whether pretreatment of osteoblasts with EGCG would protect matrix mineralization and differentiation from DEX effects. This process was assessed by Alizarin Red staining. Results in **Figure 3A** indicated that EGCG improved mineralization by osteoblasts treated with DEX. Runx2 is the most specific gene marker expressed at the earliest stage of bone formation and represents the initiation of osteoblast differentiation (Thiagarajan et al., 2017); BMP-2 plays a crucial role in bone regeneration (De La Vega et al., 2017). Osterix affects cortical bone homeostasis in bone-forming cells (Bendre et al., 2018) and could also be salvaged by EGCG, countering DEX. All the above results demonstrated that EGCG could ameliorate osteogenic differentiation hampered by DEX-induced cellular dysfunction. These findings demonstrated that EGCG enhanced the maturity of osteoblasts.

In addition, we designed an *in vivo* experiment to validate the effect of EGCG on bone in the GIO rat model. Micro-CT was applied to quantitatively represent the microarchitecture of bone geometry through a range of computed attenuation-based parameters for both *in vivo* and *ex vivo* applications (Hao et al., 2016). Additionally, since BMD is responsible for 50–70% of total bone strength, densitometry was employed as an imperative method to reflect bone quality. Microstructural parameters, such as BMD, Tb.Th, Tb.Sp, Tb.N, BV/TV, Conn.D, and SMI (Shim et al., 2016), were selected to measure the microstructure of trabecular bone. These results revealed that GSTD could improve bone quality in the GIO rat model.

EGCG is of great abundance in green tea and has high bioactivity. The high antioxidant activity of EGCG makes it beneficial for protecting the body from oxidative damage (Kim et al., 2014). Furthermore, it is currently recognized as being able to regulate the conversion of 11β-HSD, thereby reducing glucocorticoid activity. EGCG could also improve osteogenic differentiation and survival rate of osteoblasts under stress caused by DEX. On the basis of our findings, we can conclude that EGCG could be a promising candidate agent for effectively ameliorating GIO through the activation of the Nrf2/HO-1 pathway. This study offers a novel strategy for the prevention of GIO by using natural products.

## Author Contributions

SL and QF designed and planned the experiments. SL prepared the draft of manuscript. SL, LY, and SM participated in carrying out the experiments. SL and LY provided the condition for experiments, analyzed the data, and prepared the draft of the manuscript. QF conceived the idea, supervised all research, and revised the manuscript. All authors reviewed the manuscript.

## Conflict of Interest Statement

The authors declare that the research was conducted in the absence of any commercial or financial relationships that could be construed as a potential conflict of interest.
